# Neurovascular anatomy around the knee: Relevance of the dangers of self-drilling external fixator pin tips

**DOI:** 10.1051/sicotj/2019006

**Published:** 2019-03-25

**Authors:** Lucy Reipond, Alex Trompeter, Matthew Szarko

**Affiliations:** St. George’s, University of London Cranmer Terrace London SW17 0RE UK

**Keywords:** External fixation, Self drilling pin tips, Popliteal vessels, Tibial nerve

## Abstract

*Introduction*: With external fixation of the femur and tibia, iatrogenic injury to neurovasculature from self-drilling tips of fixation pins is an important consideration in pin placement. Precise knowledge of the neurovascular anatomy in the distal femur and proximal tibia is important to limit potential pin misplacement.

*Method*: Six pin placement sites on six cadaveric legs were used in accordance with current placement techniques. After pin placement, the soft tissue around each pin was dissected and the distances between the pin tips and the surrounding neurovasculature were measured.

*Results*: The resultant data allow for a description of safe and unsafe corridors which can be used for external fixator pin placement. Safe sagittal insertion into the distal femur should consist of two pins: (1) 90 mm ± proximal from the proximal pole of the patella and 3 mm ± medially, (2) 55 mm ± proximal from the proximal pole of the patella and 2 mm ± laterally. Safe coronal insertion into the distal femur should consist of two pins: (1) 30 mm ± proximal to the lateral epicondyle, (2) 100 mm ± proximal to the lateral epicondyle. Safe proximal tibial pin placement should consist of two pins and be placed at an oblique angle: (1) 20 mm ± distal to the tibial tuberosity and 2 mm ± medially, (2) 55 mm ± distal to the tibial tuberosity and 2 mm ± medially.

*Discussion*: This study forms an investigation into the safe areas for placement of external fixator pins, within the distal femur and proximal tibia, specifically, detailing the best practice for pin placement in relation to the tips of the external fixation pins.

## Introduction

External fixation (EF) is a minimally invasive technique which is used for fracture immobilization. Commonly used for open fractures, this technique provides stability and support whilst aiding in healing. Other uses of EF include limb reconstruction surgery, as well as, the treatment of osteomyelitis and non-union fractures [1, 2]. The use of EF is preferred when there is accompanying soft tissue injury, as the adaptability of the device means that it can be placed in various positions; to avoid areas of damaged tissues, allowing the management of both the fracture and the surrounding tissue. As well as providing stability, the device may also promote early exposure to gradual weight bearing, and range of movement of joints, both of which can help promote fracture healing and rehabilitation [3, 4].

Whilst both partially threaded and fully threaded EF pins only penetrate one side of the limb, their tips pass beyond the cortex of the opposing side of the bone, therefore potentially endangering nearby neurovasculature. Anatomical areas that are considered to be of low risk of iatrogenic injury to neurovascular structures (known as safe corridors) are well described for pin insertions. However, little has been discussed regarding the tips of self-drilling pins and their proximity to neurological and vascular structures. Traditional safe corridors are described for pins or wires that traverse the entire limb, and as such are often narrow. Pins inserted into only one side of the limb, often during temporary use of external fixators, may have a wider safe arc of passage on that side; however, their tips may stray into unsafe corridors on the opposite side of the bone. Whilst EF pins often push the neurovascular structures aside when inserted into a limb, and do not penetrate them, erosion through rubbing can result in bleeding or progressive numbness [5–8]. The prevalence of EF pin damage to neurovasculature on the tip-side of the pin, remains an understudied area. However, iatrogenic damage from EF pin tips have been seen to lead to pseudoaneurysms and other clinical complications such as partial or complete occlusion of arteries [5].

Hence, treatments, if they are available, will be required in order to correct these complications if and when they occur. The patient will have to undergo more surgical procedures, for which they may not be systemically or psychologically well enough, due to the traumatic experiences they have faced leading up to requiring EF. Therefore, this highlights the importance of researching the proximity of EF pin tips have to neurovascular structures.

The present study investigates how the importance of neurovascular variation can influence the risk of iatrogenic injuries caused by EF pin tips and elucidate safe corridors to reduce such injuries.

## Materials and methods

**Figure 1:**
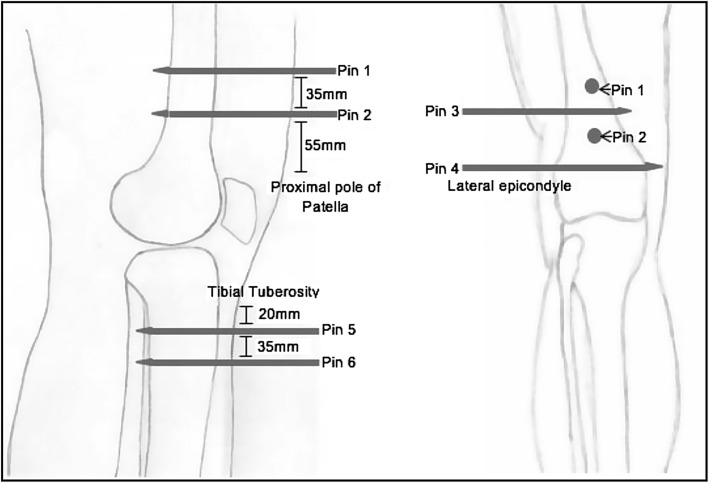
This figure illustrates the positions of the six EF pins and shows how the tips of the EF pins pass through to the other side of the bone, endangering the neurovasculature. The pins were placed specifically about the knee, within the metaphyseal/metadiaphyseal portions of the tibia/femur relative to the knee joint. The focus was on proximity that the EF pin tips obtained to the neurovascular bundle within these areas, as opposed to the stabilization constructs. The pins were inserted in orthogonal planes and perpendicular to the axes of the limb, the exact locations are described below: Pin 1 – inserted anterior to posterior within the distal femur, 90 mm ± proximal to the proximal pole of the patella.Pin 2 – inserted anterior to posterior within the distal femur, 55 mm ± proximal to the proximal pole of the patella.Pin 3 – inserted lateral to medial in the distal femur, approximately 100 mm ± proximal to the lateral epicondyle.Pin 4 – inserted lateral to medial in the distal femur, positioned 30 mm ± proximal to the lateral epicondyle.Pin 5 – inserted anterior to posterior in the proximal tibia; 20 mm ± below the tuberosity and 2 mm ± medially. This placed the pin parallel to the lateral wall of the tibia, once it had been drilled into place.Pin 6 – inserted anterior to posterior in the proximal tibia; 35 mm ± distally to pin 5.

Six EF pins (Figure 1) were inserted into the distal femur and proximal tibia of the right and left lower limbs of three cadavers, by a consultant orthopaedic trauma surgeon. None of the cadavers had limb abnormalities or previous musculoskeletal conditions. The cadavers (phenol and glycerol preserved) were obtained and utilised in accordance with the Human Tissue Act (2004).

The EF pins used were self-drilling, self-tapping 5-mm pins (DePuy-Synthes UK; and Stryker UK Limited). Standard fixator constructs were applied using self-drilling pins as detailed in the operative technique of the Manufacturer (Hoffman III fixator, Stryker, Mahwah, NJ, USA). All pins had a similar geometry, with self-drilling tips extending 5–8 mm beyond the threaded portion and were inserted using an electric drill.

The positions of the pins were checked with fluoroscopic imaging (a General Electric stenoscope), in accordance with an Ionising radiation medical exposures regulations (IRMER)-certified trained technician, to ensure uniform pin placement in all limbs and that the EF pins penetrated fully through the far cortex.

Dissections were undertaken of the distal thigh and proximal leg, in order to measure the proximity the EF pins had to important neurovasculature. Pins 1, 2, 5 and 6 were inserted into the limbs within the sagittal plane; thus, within the sagittal plane, the neurovasculature was located posterior to the tips of the pins. This, therefore, resulted in medial and lateral pin localities within the coronal plane. Pins 3 and 4 traversed the posterior distal thigh; hence, the neurovasculature was located in the sagittal plane, lying posterior to the tips of the pins.

Four measurements were taken of pins 1, 2, 5 and 6. These measurements recorded the sagittal and coronal distances, which the neurovasculature obtained from the tips of the EF pins, when the knee joints were extended (0°) and flexed (45°). Furthermore, two measurements were noted of pins 3 and 4; the sagittal distance measured when the knee joints were extended (0°) and flexed (45°). Measurements were all undertaken using Vernier digital callipers (+0.01 mm). The average distances were then calculated from the right and left legs of the three cadavers.

## Results

**Figure 2:**
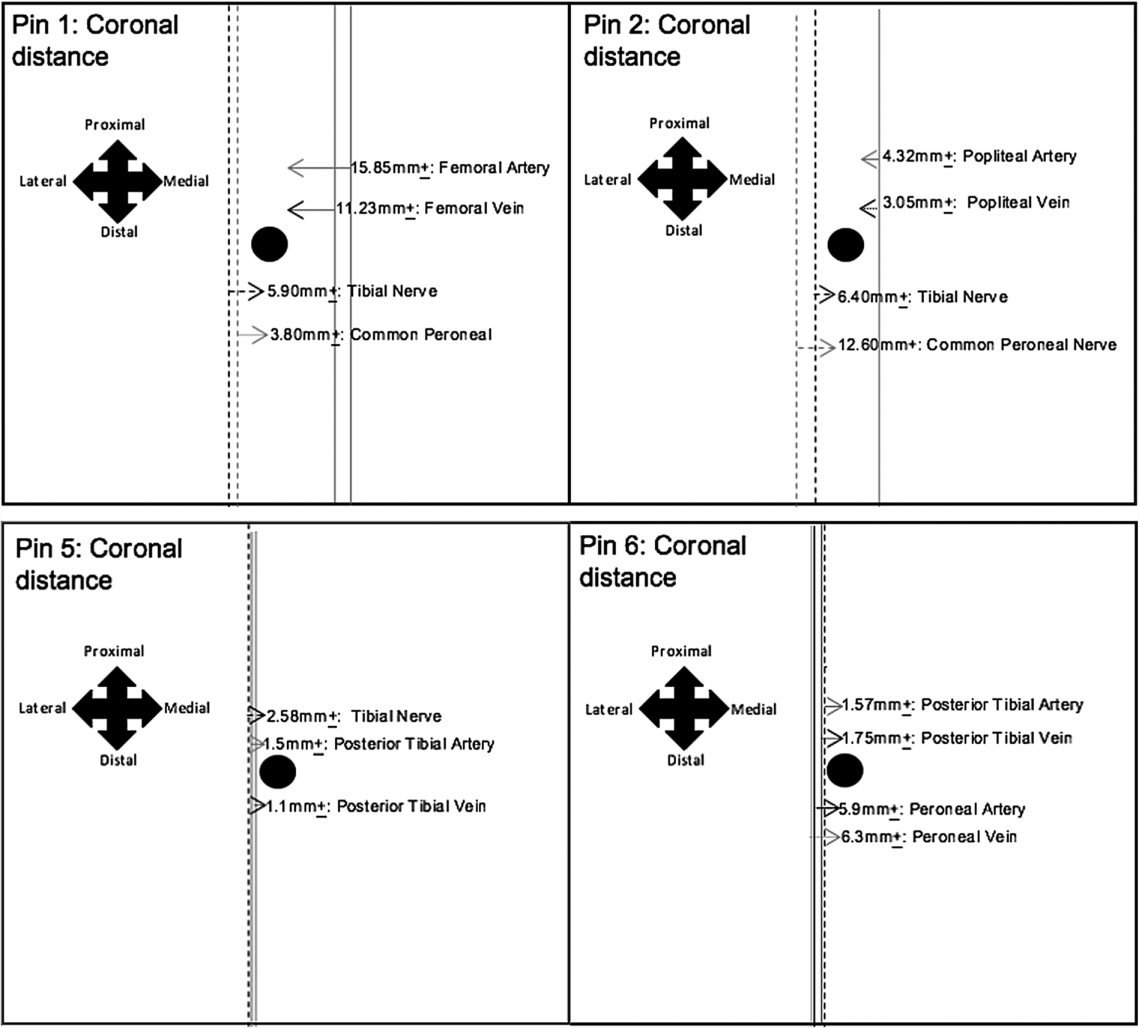
The results show that the tips of EF pins 1 and 2 attained a close proximity to the large neurovasculature, within the coronal plane, specifically; the tibial and common peroneal nerve and the femoral/popliteal vessels, respectively. Moreover, the measurements obtained for the average coronal distances, between the tibial nerve and pin 2, differed within the left and right distal thighs; highlighting neurovascular variation. Additionally, the tips of pins 5 and 6 also attained a close proximity to the large neurovasculature; as shown in the figure. Therefore, it would be beneficial to modify the locations of EF pins 1, 2, 5 and 6, in order to reduce the risk of iatrogenic injury to these vessels.

**Figure 3:**
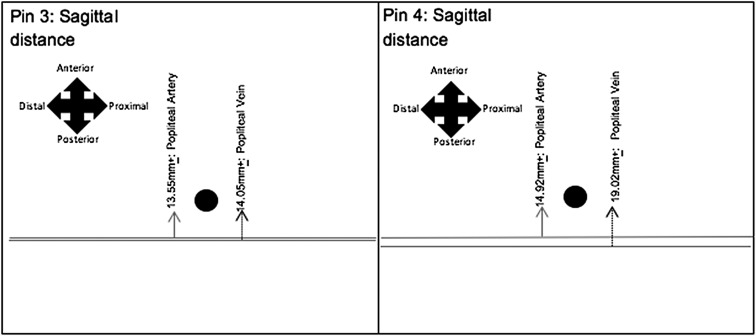
The results obtained for the average sagittal distances between the tips of EF pins 3 and 4, achieved a suitable distance, ranging from 13.55 mm ± 1.09 mm to 19.01 mm ± 1.20 mm; resulting in a lower risk of iatrogenic injury.

Figures 2 and 3 represent the average distances between the tips of EF pins and the principal neurovasculature, in relation to the sagittal and coronal planes. The black circle symbolises the EF pin tip, with the lines representing the neurovasculature.

## Discussion

The prevalence of external fixation has continually increased to be one of the primary techniques used to treat even the most complicated of cases, for example in emergency stabilisation of patients with life-threatening injuries, limb reconstruction procedures and definitive fracture management [2]. The safety corridors of EF related to pin insertion are relatively well known. Within the area surrounding pins 1–4 (distal femur), the Sartorius muscle, which originates from the anterior superior iliac spine, descends laterally across the thigh and inserts into the medial femoral condyle. This muscle marks the pathway of the neurovascular bundles, as well as providing protection for it. Consequently, the safety corridors within the distal thigh spans approximately 220° clockwise, beginning from the lateral boarder of the Vastus Lateralis [9,12]. In regard to the lateral distal thigh, avoiding Hunter’s canal and subsequently the neurovascular bundle is crucial. Moreover, the safety corridors surrounding the location of pins 5 and 6 (proximal tibia) for pin placement, are said to be “either in a sagittal plane medial to the tibial crest or perpendicular to the anteromedial surface of the tibia” [12].

However, there is limited research regarding the proximity that the tips of the EF pins have to the neurovascular structures, once inserted into the bone, despite the fact that iatrogenic injury caused by self-drilling pin tips can lead to serious complications [5,13].

### Impact of flexion of the knee joint

During insertion of EF pins, flexing the knee joint is a well-known technique which is used to increase the distance, sagittally, between the tips of EF pins and the neurovasculature. This is because, as the knee joint is flexed, the neurovasculature moves away from the posterior surface of the associated bone. This was the case when comparing the distances between the tips of EF pins and the neurovasculature during extension. However, after insertion of the EF pins, the knee joint is normally extended to secure the pins into a frame. Extending the knee joint could potentially move the neurovasculature closer to the EF pin tips. Therefore, it is essential that the positions of the EF pins are at a suitable distance from the neurovasculature in both flexion and extension.

### Femoral fixators (pins 1–4)

In a clinical setting, a two-plane fixator frame is preferred when treating a femoral fracture. The pins are placed within the sagittal plane, as well as in the coronal plane, from the lateral aspect of the femur. This aids neutralization of the sagittal forces that are exerted on the thigh, caused by the muscular structures that attach. This configuration is demonstrated by the positions of pins 1–4 [9].

The average measurements obtained between pin 1 and the vessels indicate that it would be beneficial to reposition the EF pin 3 mm medially within the anterior femur, in order to increase the distance between the tibial and common peroneal nerve, hence decreasing the risk of iatrogenic injury to the neurovasculature. Additionally, it would be beneficial to modify the location of the second pin, 2 mm laterally, as there was a larger distance between the tibial nerve (TN) and the common peroneal nerve to pin 2, and a smaller distance to the popliteal vessels. A study by Kishan et al. [9] also concluded that the optimal position of an EF pin, when treating a distal femoral fracture, is in the anterolateral aspect of the thigh, thus supporting the conclusion of modifying the position of the second pin.

Furthermore, the measurements obtained for the average coronal distances between the neurovasculature and pin 2 differed within the left and right distal thighs. The TN was shown to convey a more lateral pathway, on average, in the posterior aspect of the left distal thighs, compared to the average distances obtained for the right distal thighs. This anatomical variation emphasises the potentially increased risk of iatrogenic injury caused by the tips of EF pins. Variations that can occur in either the right or left lower limb may lead to the vessels conveying a pathway that enters into the so-called “safety corridors”, consequently increasing the risk of injury.

### Lateral to medial placement of EF pins within the distal femur

A unilateral frame can also be used as a temporary device; this involves placing the pins within the lateral aspect of the thigh (position of pins 3 and 4). As the EF pins are only located in one plane, the EF device may lack mechanical stability. Therefore, other factors such as pin size and spread have to be manipulated accordingly, to ensure the device has sufficient mechanical stability [9, 10].

The neurovasculature that are at risk when inserting the third and fourth pins into the distal femur, are the vessels passing through Hunter’s canal, specifically, the popliteal artery and the popliteal vein. However, the distances measured from the tips of pin 3 and pin 4 to the neurovasculature, sagittally, consistently achieved a suitable distance. As a result, the position of both pins can be concluded to be within a safe distance of the associated neurovascular structures, and can be used to stabilise femoral fractures safely, or aid the stabilisation of tibial fractures.

### Tibial fixators (pins 5 and 6)

Sagittally placed pins are commonly used to treat proximal tibial fractures. The position of the pins counteracts the forces that would normally be placed upon the leg, such as anteroposterior and transverse bending movements, therefore, maintaining stability of the fractured bone [3, 11]. However, in this study, the average measurements obtained for the coronal and sagittal distances between the tips of pins 5 and 6 and the principal neurovasculature (posterior tibial artery, posterior tibial vein, peroneal artery, peroneal vein, TN and common peroneal nerve) were extremely small; thus, the locations of pins 5 and 6 carry a high risk of causing iatrogenic injury to the neurovasculature. Therefore, to decrease the risk of injury, it would be beneficial to insert the EF pins into the tibia at an oblique angle, resulting in an increased distance, coronally, between the tips of the pins and vessels.

**Figure 4:**
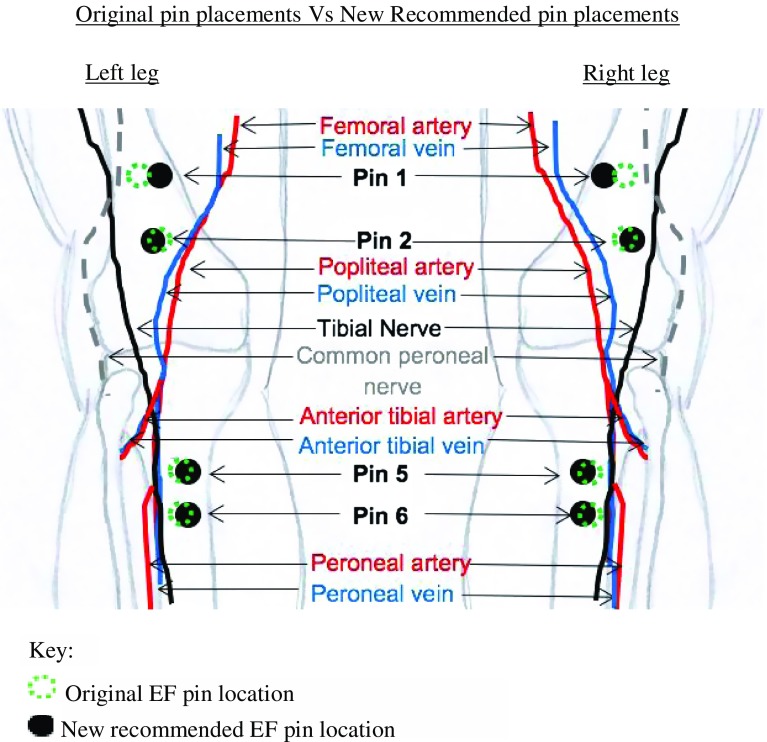
This figure represents the original locations of the EF pins 1, 2, 5 and 6 and compares these data against where the pins should be placed, after analysing the results. The recommended locations of EF pins 1, 2, 5 and 6 are described below: Pin 1 – inserted anterior to posterior within the distal femur, 90 mm ± proximal to the proximal pole of the patella and 3 mm ± medially.Pin 2 – inserted anterior to posterior with the distal femur, 55 mm ± proximal to the proximal pole of the patella and 2 mm ± laterally.Pin 5 – inserted anterior to posterior in the proximal tibia; 20 mm ± distal to the tibial tuberosity and 2 mm ± medially, at an oblique angle.Pin 6 – inserted anterior to posterior in the proximal tibia, 35 mm ± distally to pin 5 and 2 mm ± medially, at an oblique angle.

Therefore, after analysis of the resultant data, recommended positions of the EF pins within the distal femur and proximal tibia are shown in Figure 4.

## Conclusion

When EF pins are inserted into the well-known safety corridors, there is still a high incidence of EF pin tips being in close proximity to neurovascular structures. Knowledge of neurovasculature pathways, and any diversifications within them, is critically important. The neurovascular pathways are highly variable and can potentially increase the risk of iatrogenic injuries when inserting EF pins [5,13].

Consequently, this led to the modification of the positions of EF pins 1, 2, 5 and 6. Pins 3 and 4 were shown to obtain a safe distance from the neurovascular structures; thus, their positions were not changed. In summary, the recommended positions of all the EF pins concluded in this article are: safe sagittal placed pins within the distal femur should include two pins (1) 90 mm ± proximal from the proximal pole of the patella and 3 mm ± medially, (2) 55 mm ± proximal from the proximal pole of the patella and 2 mm ± laterally. Safe coronal insertion into the distal femur should consist of two pins: (1) 30 mm ± proximal to the lateral epicondyle, (2) 100 mm ± proximal to the lateral epicondyle. Safe proximal tibial pin placement should consist of two pins and be placed at an oblique angle: (1) 20 mm ± distal to the tibial tuberosity and 2 mm ± medially, (2) 55 mm ± distal to the tibial tuberosity and 2 mm ± medially.

Therefore, the results from this article highlight the need for dedicated and continued research to precisely determine the variations in neurovascular pathways, in order to decrease the risk of iatrogenic injury to the patient.
